# Treatment Emergent Dolutegravir Resistance Mutations in Individuals Naïve to HIV-1 Integrase Inhibitors: A Rapid Scoping Review

**DOI:** 10.3390/v15091932

**Published:** 2023-09-15

**Authors:** Kaiming Tao, Soo-Yon Rhee, Carolyn Chu, Ava Avalos, Amrit K. Ahluwalia, Ravindra K. Gupta, Michael R. Jordan, Robert W. Shafer

**Affiliations:** 1 Division of Infectious Diseases, Department of Medicine, Stanford University, Stanford, CA 94305, USA; kmtao@stanford.edu (K.T.);; 2 Department of Family and Community Medicine, University of California San Francisco, San Francisco, CA 94011, USA; 3 Careen Center for Health, Gaborone, Botswana; 4 Tufts University School of Medicine, Boston, MA 02111, USA; 5 Cambridge Institute of Therapeutic Immunology and Infectious Disease (CITIID), Cambridge CB2 0AW, UK; 6 Division of Geographic Medicine and Infectious Diseases, Tufts Medical Center, Boston, MA 02111, USA

**Keywords:** Dolutegravir, HIV-1 integrase, antiviral resistance, mutations

## Abstract

**Background**: Dolutegravir (DTG)-based antiretroviral therapy (ART) rarely leads to virological failure (VF) and drug resistance in integrase strand transfer inhibitor (INSTI)-naïve persons living with HIV (PLWH). As a result, limited data are available on INSTI-associated drug resistance mutations (DRMs) selected by DTG-containing ART regimens. **Methods:** We reviewed studies published through July 2023 to identify those reporting emergent major INSTI-associated DRMs in INSTI-naïve PLWH receiving DTG and those containing in vitro DTG susceptibility results using a standardized assay. **Results:** We identified 36 publications reporting 99 PLWH in whom major nonpolymorphic INSTI-associated DRMs developed on a DTG-containing regimen and 21 publications containing 269 in vitro DTG susceptibility results. DTG-selected DRMs clustered into four largely non-overlapping mutational pathways characterized by mutations at four signature positions: R263K, G118R, N155H, and Q148H/R/K. Eighty-two (82.8%) viruses contained just one signature DRM, including R263K (*n* = 40), G118R (*n* = 24), N155H (*n* = 9), and Q148H/R/K (*n* = 9). Nine (9.1%) contained ≥1 signature DRM, and eight (8.1%) contained just other DRMs. R263K and G118R were negatively associated with one another and with N155H and Q148H/K/R. R263K alone conferred a median 2.0-fold (IQR: 1.8–2.2) reduction in DTG susceptibility. G118R alone conferred a median 18.8-fold (IQR:14.2–23.4) reduction in DTG susceptibility. N155H alone conferred a median 1.4-fold (IQR: 1.2–1.6) reduction in DTG susceptibility. Q148H/R/K alone conferred a median 0.8-fold (IQR: 0.7–1.1) reduction in DTG susceptibility. Considerably higher levels of reduced susceptibility often occurred when signature DRMs occurred with additional INSTI-associated DRMs. **Conclusions:** Among INSTI-naïve PLWH with VF and treatment emergent INSTI-associated DRMs, most developed one of four signature DRMs, most commonly R263K or G118R. G118R was associated with a much greater reduction in DTG susceptibility than R263K.

## 1. Introduction

Among integrase strand transfer inhibitor (INSTI)-naïve persons living with HIV (PLWH), antiretroviral (ARV) therapy (ART) with the second-generation INSTIs dolutegravir (DTG) and bictegravir has been associated with low rates of virological failure (VF) and emergent INSTI-associated drug resistance mutations (DRMs) [1]. In the past five years, DTG has been particularly instrumental in the World Health Organization (WHO)’s efforts to control the HIV-1 pandemic. DTG is now recommended in multiple clinical situations, including for first-line ART, programmatic transition for individuals previously treated with a first-line nonnucleoside reverse transcriptase (RT) inhibitor (NNRTI)-based regimen, second-line ART following the VF of a first-line NNRTI-based regimen, and third-line ART in combination with the ritonavir-boosted protease inhibitor (PI) darunavir (b/DRV) [2].

Given that resistance to DTG is determined genotypically, we conducted a rapid review of all studies reporting the emergence of INSTI-associated DRMs in INSTI-naïve PLWH experiencing VF while receiving a DTG-containing regimen and of in vitro susceptibility data associated with DTG-selected DRMs. Our group previously published a systematic review of DTG-selected DRMs in 2019 that summarized data from 14 publications reporting 29 cases of DTG-selected DRMs in previously INSTI-naïve PLWH and 12 publications reporting the results of in vitro DTG susceptibility testing using the PhenoSense assay for 179 distinct HIV-1 isolates [3]. In the ensuing four and a half years, there has been a more than three-fold increase in the reported instances of emerging DTG resistance and an approximately 50% increase in the number of in vitro DTG susceptibility test results. 

## 2. Methods

We reviewed PubMed using the search phrase “(Dolutegravir) AND ((“Drug Resistance”[Mesh] OR Resistance) OR “Clinical Trial” [Publication Type] OR (“Mutation”[Mesh] OR Mutation(s)) OR (“in vitro susceptibility”))”, last updated July 18, 2023, to identify studies containing data pertinent to the following two types of information: (1) integrase mutations emerging in INSTI-naïve persons receiving DTG; and (2) the effect of integrase mutations on in vitro DTG susceptibility.

We included studies that provided complete integrase sequences as well as studies that reported only those integrase mutations noted by study authors. Nonpolymorphic major INSTI-associated DRMs were defined as H51Y, T66A/I/K, E92G/Q, G118R, F121Y, E138A/K/T, G140A/C/S, Y143C/H/R/S, S147G, Q148H/R/K, S153Y/F, N155H, S230R, and R263K [4]. Additional polymorphic and minor nonpolymorphic DRMs including A49G, M50I, L74I/M, V75A, Q95K, T97A, G149A, V151I, E157Q, G163R/K, and D232N were noted when they occurred in combination with one or more major nonpolymorphic DRMs. 

When HIV-1 isolates containing INSTI-associated DRMs were reported in more than one publication describing the results from a single clinical trial or cohort, we linked the isolates to just one of the publications. For the few PLWH with more than one isolate containing an INSTI-associated DRM, we recorded the isolate containing the largest number of DRMs. When sequences were available, we analyzed them for their sequence quality and for the presence of APOBEC-mediated G-to-A hypermutation. Two sequences, including one with multiple sequencing errors and another with G-to-A hypermutation, were excluded from analysis. 

To determine the effect of INSTI-associated DRMs on DTG susceptibility, we analyzed all published in vitro DTG susceptibility results performed using the PhenoSense assay because this is the most commonly used assay and because it is highly reproducible [5]. Reductions in DTG susceptibility using this assay have also been previously correlated with virological outcome [6,7]. Redundant viruses obtained from the same individual and isolates containing mixtures at signature DTG-associated DRM positions were excluded from analyses of in vitro susceptibility. 

If a tested HIV-1 isolate was a site-directed mutant and the list of mutated integrase residues was provided, the isolate’s mutation list was considered to be complete. If a tested HIV-1 isolate was derived from a clinical sample, the mutation list was considered to be complete only if the nucleic acid sequence or a complete list of amino acid differences from a reference sequence was provided. Replication capacity values were obtained from published studies of isolates in the Stanford HIV Drug Resistance Database (HIVDB) that underwent susceptibility testing using the PhenoSense method [8]. Replication capacity was defined as the extent of virus replication during a single round of replication compared with a wildtype reference isolate in the absence of ARV drugs. 

We summarized the results for the most common patterns of INSTI-associated DRMs and performed a least squares regression (LSR) analysis in which DRMs were included as explanatory variables and the log fold change in susceptibility was the response variable. Ten repetitions of 5-fold cross-validation were performed to estimate the variance among the fitted coefficients. 

We also created a list of positively correlated mutation pairs defined as those (1) co-occurring in at least two individuals, (2) having a nonparametric Spearman’s correlation coefficient (ρ) ≥ 0.20, and (3) having a *p* value ≤ 0.05. For this analysis, mutations present as part of a mixture with wildtype were excluded. 

## 3. Results

### 3.1. Search Results

**Figure 1:**
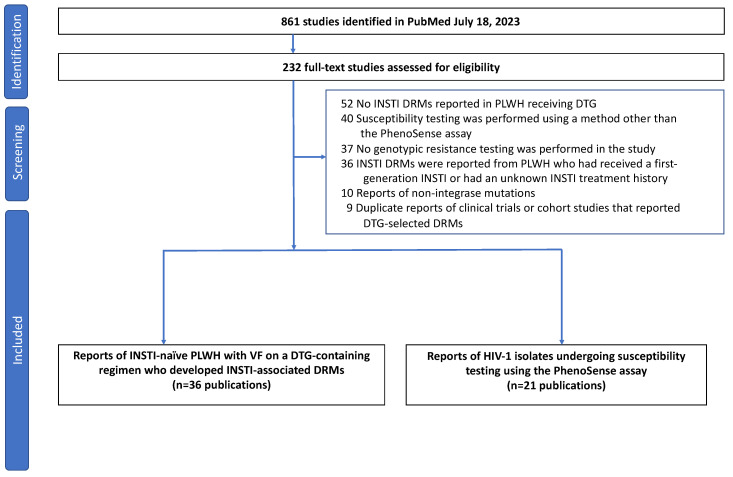
Flow chart summarizing the review process. One of the 36 publications describing emergent INSTI-associated mutations was identified through a routine BLAST search of GenBank as part of the work for maintaining the Stanford HIV Drug Resistance Database and through the review of references in other papers with emergent INSTI-associated DRMs selected by DTG. Of the 21 publications reporting DTG susceptibility testing, 2 were reported only at scientific meetings. These were retrieved from the Stanford HIV Drug Resistance Database (https://hivdb.stanford.edu/cgi-bin/Phenotypes.cgi?Gene=IN; last accessed 18 July 2023) and cited in our earlier review [3]. Abbreviations: VL (virus load); INSTI (integrase strand transfer inhibitor); DRMs (drug-resistance mutations) VF (virological failure); PLWH (people living with HIV-1).

As of 18 July 2023, 861 publications were retrieved from PubMed (Figure 1). Following the review of their titles and abstracts, 232 publications were submitted for full-text review. Following full-text review, 36 publications were found to have reports of INSTI-naïve PLWH who developed one or more INSTI-associated DRMs while receiving DTG [9,10,11,12,13,14,15,16,17,18,19,20,21,22,23,24,25,26,27,28,29,30,31,32,33,34,35,36,37,38,39,40,41,42,43,44], and 21 publications contained in vitro susceptibility results performed using the PhenoSense assay [32,33,35,45,46,47,48,49,50,51,52,53,54,55,56,57,58,59,60,61,62].

### 3.2. Reports of Emergent INSTI-Associated DRMs

**Figure 2:**
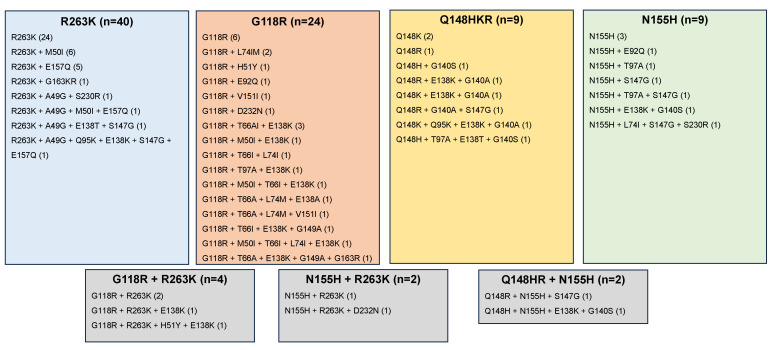
Patterns of INSTI-associated DRMs occurring in 99 previously INSTI-naïve individuals with virological failure on a DTG-containing regimen who developed a major nonpolymorphic INSTI-associated DRM defined as one of the following mutations: H51Y, T66A/I/K, E92G/Q, G118R, F121Y, E138A/K/T, G140A/C/S, Y143C/H/R/S, S147G, Q148H/R/K, S153Y/F, N155H, S230R, and R263K. Polymorphic and accessory DRMs were identified only when they occurred in an isolate that also contained a major nonpolymorphic INSTI-associated DRM. Additional patterns of mutations that do not conform to the above categories included one with and eight without signature DRMs. The one with signature DRMs included L74I + G118GR + E138K + Q148QR + R263RK. The eight without signature mutations included S230R (*n* = 3), T66I, E138K, H51Y + S147G, E138A + G149A, and E138K + D232N.

Thirty-six publications reported 99 one-per-person viruses with one or more major nonpolymorphic INSTI-associated mutations from a previously INSTI-naïve individual treated with a DTG-containing regimen (Table 1). Eleven publications reported 40 PLWH in clinical trials. Five publications reported 7 PLWH in clinical cohorts. Nine publications reported 38 PLWH in case series. Twelve publications reported 14 PLWH in case reports. The complete integrase sequence was available for 17 isolates reported in five studies. Figure 2 depicts the complete list of one-per-person mutation patterns.

The PLWH developing INSTI-associated DRMs included 49 (49.5%) ART-experienced viremic individuals receiving DTG plus two NRTIs, 11 (11.1%) ART-experienced individuals with virological suppression receiving DTG monotherapy, 15 ART-naïve viremic individuals receiving DTG plus two NRTIs or DTG plus 3TC, and 24 individuals with other combinations of ART history, virological status, and DTG ART (Table 2). The geographic locations included Europe (35%), Sub-Saharan Africa (35%), North America (12%), and Brazil (5%). An additional 13% of individuals were participants in multinational clinical trials. 

DTG-selected INSTI-associated DRMs clustered into four largely non-overlapping mutational pathways characterized by amino acid mutations at four signature positions: (1) R263K; (2) G118R; (3) N155H; and (4) Q148H/R/K. Indeed, 82 (82.8%) of 99 virus sequences contained just one of the signature mutations including R263K (*n* = 40), G118R (*n* = 24), N155H (*n* = 9); and Q148H/R/K (*n* = 9). Nine virus sequences contained more than one signature DRM including two sequences in which a mixture was present at one or more of the signature positions. Eight sequences contained just other (i.e., non-signature) INSTI-associated DRMs including S230R (*n* = 3), T66I, E138K, H51Y + S147G, E138A + G149A, E138K + D232N.

Q148 mutations and N155H were over-represented in the 11 PLWH with virological suppression receiving DTG monotherapy. Eight individuals in this category developed a Q148 mutation and/or N155H, while just two developed R263K. No difference was observed in geographic region, past ART history, virological status, and DTG ART between those developing R263K compared with G118R. 

R263K was reported more commonly with E157Q and A49G than with other DRMs (Table 3). G118R was reported more commonly with T66A, L74MI, and E138K. Q148 mutations were significantly associated with mutations at position 140 and S147G. N155H mutations were significantly associated with S147G, T97A, and G140S. G118R and R263K were negatively correlated with each other and with Q148 mutations and N155H. 

Although sequences were not available from any individual prior to receiving DTG, it was possible to infer from the available post-DTG-containing sequences that R263K resulted from a single G→A transition, while the remaining signature DRMs usually resulted from a single nucleotide transversion. Nearly all previously published wildtype sequences indicate that position 118 is usually encoded by GGC/T (rather than GGG/A), regardless of subtype (https://hivdb.stanford.edu/cgi-bin/Probe.cgi, last accessed on 18 July 2023). Therefore, G118R would be expected to result from a single G→C transversion at its first nucleotide rather than by double nucleotide mutations at its first and third codon positions. Indeed, of the eight isolates containing G118R for which sequences were available, seven were encoded by CGC or CGT at position 118, suggesting that these resulted from a G→C transversion at its first codon position. 

### 3.3. Phenotypic Impact of DTG-Selected INSTI-Associated DRMs

There were 21 studies reporting 269 non-redundant in vitro susceptibility results performed using the PhenoSense assay. These results included 151 site-directed mutants and 118 clinical isolates (Supplementary Table S2). For 206 (77%) of the isolates, complete mutations lists were available, while for 63 (23%), only those DRMs reported by authors were available. 

R263K alone conferred a median 2.0-fold reduction in DTG susceptibility (IQR: 1.8–2.2; range: 1.5–3.3) (Table 4). With ≥1 additional DRM, it conferred a median 3.2-fold reduction in susceptibility (IQR: 1.8–5.8; range: 1.3–7.0). G118R alone conferred a median 18.8-fold reduction in DTG susceptibility (IQR: 14.2–23.4; range: 9.6–28). With ≥1 additional DRM, it conferred a median 19.0-fold (IQR: 11.8–28.5; range: 7.2–52) reduction in susceptibility. N155H alone conferred a median 1.4-fold reduction in DTG susceptibility (IQR: 1.2–1.6; range: 1.1–2.1). With ≥1 additional DRM, it conferred a median 2.0-fold (IQR: 1.5–2.4; range: 1.1–68) reduction in susceptibility. Q148H/R/K alone conferred a median 0.8-fold reduction in DTG susceptibility (IQR:0.7–1.1; range: 0.4–1.6). With ≥1 additional DRM, they conferred a median 4.1-fold (IQR: 2.2–8.7; range: 0.5–186) reduction in susceptibility.

**Figure 3:**
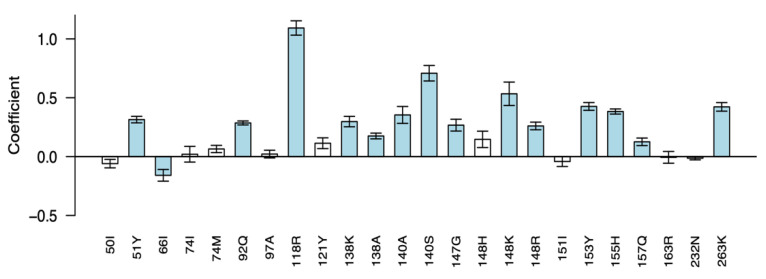
Regression coefficients of the least-squares regression (LSR) model for predicting fold reductions in DTG susceptibility using genotypic predictors. Integrase mutations scored by the HIVDB drug resistance interpretation system were included as explanatory variables and log fold change in susceptibility was the response variable. Ten repetitions of 5-fold cross-validation were performed to estimate the variance among the fitted coefficients. The mutations shown are those that occurred at least 5 times in isolates that underwent susceptibility testing using the PhenoSense assay. For each mutation, the y axis indicates the magnitude of the mean coefficient of 50 LSR runs (10 × 5-fold cross-validation), and the error bar indicates the standard deviation from the mean. Positive coefficients indicate mutations that reduced DTG susceptibility. Negative coefficients indicate mutations that increased DTG susceptibility. Bars representing coefficients whose cross-validated means were ≥1.5-fold and ≥3 standard deviations from zero are blue; other coefficient bars are white, indicating a lack of statistical significance after cross-validation.

Of the DRMs included in the least-squares regression model, 26 mutations occurred ≥5 times in the dataset (Figure 3). The regression coefficients of 14 mutations were associated with ≥1.5-fold reduced susceptibility including five signature DTG mutations at four positions, G118R, Q148R/K, N155H, R263K, and eight additional nonpolymorphic INSTI-associated DRMs, including H51Y, E92Q, E138A/K, G140A/S, S147G, and S153Y.

**Figure 4:**
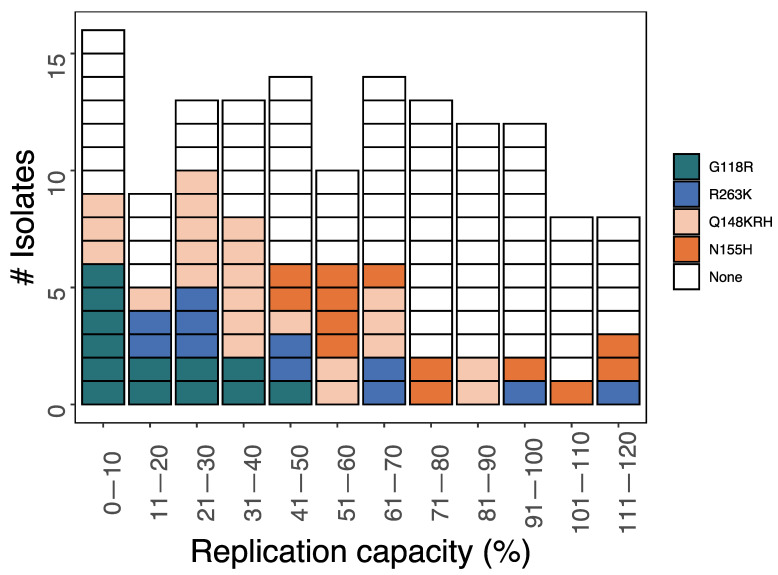
Replication capacities of 60 HIV-1 isolates containing a signature DTG-associated DRM and 82 HIV-1 isolates lacking such a DRM. Each of the signature DRMs were present both alone and in combination with other DRMs. For some isolates, the complete list of accompanying integrase DRMs was not available. Isolates containing more than one signature DRM are not shown.

Replication capacity results were available for 142 HIV-1 isolates, including 60 with a single signature DTG-associated DRM: G118R (*n* = 13), Q148H/R/K (*n* = 23), N155H (*n* = 13), and R263K (*n* = 11) (Figure 4). The median replication capacity was 18% for G118R (IQR: 8–30%; range: 0.3–43%), 49% for R263K (IQR: 26–66%, range: 11–115%), 35% for Q148H/R/K (IQR: 26–55%; range: 1.3–88%), and 62% for N155H (IQR:53–93%; range: 41–119%). Given the limited number of isolates with replication capacity results compared to those with DTG susceptibility results, we could not ascertain how accessory mutations influenced replication capacity. The complete list of replication capacity results and their associated integrase mutations is in Supplementary Table S2. 

## 4. Discussion

The primary objectives of this study were to identify the INSTI-associated DRMs emerging in previously INSTI-naïve persons with VF while receiving a DTG-containing regimen and to estimate the impact of these DRMs on in vitro DTG susceptibility. Among the 99 individuals reported in 36 publications who developed INSTI-associated DRMs while receiving DTG, more than 90% developed one or more of four signature INSTI-associated DRMs, including R263K, G118R, N155H, and Q148H/R/K. R263K and G118R represented distinct non-overlapping mutational pathways, as they were unlikely to occur with each other or with N155H and Q148H/R/K. 

Emergent DTG resistance was reported in 40 PLWH in 11 clinical trials for which the total number of individuals receiving DTG was known. However, most individuals with emergent DTG resistance were reported in uncontrolled studies for which the total number of individuals receiving DTG was not known. Additionally, our search identified only those studies that contained cases of emergent DTG resistance and did not identify studies in which DTG was received, but INSTI-associated DRMs did not arise. For these two reasons, a different systematic review would be required to determine the prevalence of emergent DTG resistance in different clinical scenarios.

R263K is the only signature mutation that results from a single nucleotide transition (i.e., changes between A and G and between C and T). Transitions arise more readily in viruses in general and in HIV-1 in particular [64]. In contrast, each of the remaining signature DRMs, including G118R, results from a transversion. Moreover, R263K represents a conservative amino acid change, while each of the other signature DRMs, particularly G118R, involves the substitution of an amino acid with markedly different biochemical properties compared with wildtype. Perhaps not surprisingly, G118R conferred a much greater reduction in DTG susceptibility than R263K and a lower replication capacity than each of the other signature DRMs.

The different mutational pathways can be explained in part by the reduced replication capacity and integration by viruses containing certain combinations of signature DTG-associated mutations. R263K is associated with reduced strand transfer activity and reduced infectiousness in cell culture when it occurs in combination with several of the DRMs associated with the first-generation INSTIs including Q148R [65]. In addition, the combination of G118R plus R263K is associated with reduced DNA binding, viral infectivity, and replicative capacity compared to viruses containing single mutations [66]. Conversely, two mutations which co-occurred, R263K and E157Q, have previously been shown to be synergistic in that E157Q compensates for the reduced activity of R263K-containing integrase [67].

Our in vitro susceptibility results are consistent with published biochemical data showing that DTG dissociates more rapidly from integrase DNA complexes containing G118R than those containing R263K [32]. Homology modeling demonstrates that G118R can prevent DTG and other INSTIs from binding to integrase by occluding its catalytic binding site [32]. In contrast, modeling studies suggest that R263K has a more subtle effect that involves changing the orientation of viral DNA during integration.

There are few data on the clinical significance of reduced DTG susceptibility. Data from the VIKING trials evaluated the clinical efficacy of 50 mg DTG twice daily plus an optimized background regimen on treating ART-experienced PLWH with VF and INSTI resistance after receiving a first-generation INSTI [6,7]. Among trial participants with isolates displaying <4-fold, 4 to 10-fold, and >10-fold reduced susceptibility, plasma HIV-1 RNA reductions below 50 copies/mL were attained in 76%, 54%, and 27%, respectively [7]. These findings, however, were based on data from a heavily ART-experienced population that primarily had Q148 mutations rather than G118R or R263K. Moreover, the PLWH in these trials were treated with 50 mg DTG twice daily while all of the individuals in this study were treated with 50 mg once daily. Whether the dose of DTG or its levels within an individual may influence the selection of INSTI-associated DRMs has not been studied.

Other than the increased likelihood of Q148H/R/K and N155H developing in PLWH receiving DTG monotherapy, we were unable to determine which clinical factors (e.g., ART history, presence of viremia, nature of DTG ART) were associated with particular signature DRMs. It was also not possible to determine whether HIV-1 subtype influenced which signature DRMs emerged because the absence of sequences from more than 80% of individuals made it impossible to determine the subtype of most viruses with INSTI-associated DRMs.

The presence of four mutational pathways associated with DTG resistance suggests that point-of-care assays for detecting mutations at four signature DRM positions might be useful in identifying which PLWH with VF while receiving DTG have developed DRMs that might preclude a response to continued DTG ART and adherence counseling [68]. However, despite the strong interest in such assays, none appear close to commercial development.

The second-generation INSTI cabotegravir has been reported to select for three of the signature DRMs selected by DTG: Q148R, N155H, and R263K. However, the frequency of these mutations differs from those selected by DTG. Of 18 PLWH with emergent INSTI-associated DRMs while on cabotegravir, 11 had Q148R, six had N155H, one had R263K, and none had G118R [69]. Comparable data for bictegravir is scarce due to its more limited global usage and because reports of emergent INSTI-associated DRMs in PLWH receiving bictegravir are exceptionally rare.

Two mechanisms of INSTI resistance involving mutations outside of integrase have been reported, including one caused by mutations in and near the 3′ polypurine tract (3′PPT) and one caused by mutations in the envelope glycoprotein. 3′PPT mutations have been selected during in vitro passage experiments with DTG [70] and have been shown to reduce DTG susceptibility [71] by enabling HIV-1 to produce infectious viruses from 1-LTR circles, thus bypassing integration [72]. The frequency of such mutations in persons with VF while receiving DTG has not been studied, but preliminary data suggest that they are not common [73,74,75]. Envelope mutations that increase the efficiency of cell-to-cell viral spread have been shown to reduce HIV-1 susceptibility to multiple ARV drug classes in multi-cycle replication assays but do not appear to be specific to INSTIs [76,77].

In conclusion, our review provides an initial assessment of the patterns of INSTI-associated DRMs that have been reported in INSTI-naïve PLWH receiving DTG. Future studies in low and middle income countries with rapidly expanding access to DTG-containing ART are required to determine the risk of VF and of emergent DTG resistance in the clinical scenarios in which DTG-containing regimens will most commonly be used. Additionally, studies are required to determine the clinical significance of emergent DTG resistance, particularly when arising from DRMs that are not associated with high levels of reduced DTG susceptibility. 

## Figures and Tables

**Table 1:** Reports of Emergent Major INSTI-Associated DRMs in Previously INSTI-Naïve PLWH Receiving a DTG-Containing ART Regimen.

Author (Year) ^1^	Countries	Population ^2^	ART Hx	VL Status	ART Regimen ^3^	# with DRMs
Blanco (2018) [13]	Spain	Trial	Experienced	VS	DTG	2
Cahn (2013) [9]	Multinational	Trial	Experienced	Viremic	DTG + OBR	5
Chinula (2023) [39]	Multinational	Trial	Naïve	Viremic	DTG + 2 NRTIs	1
Hocqueloux (2019) [20]	France	Trial	Experienced	VS	DTG	2
Paton (2022) [29]	Sub-Saharan Africa	Trial	Experienced	Viremic	DTG + 2 NRTIs	9
Taiwo (2018) [18]	U.S.	Trial	Naïve	Viremic	DTG + 3TC	1
Turkova (2021) [25]	Multinational	Trial	Experienced	Viremic	DTG + 2 NRTIs	4
Underwood (2022) [32]	Multinational	Trial	First-line VF	Viremic	DTG + 2 NRTIs	6
Vavro (2022) [35]	U.S.	Trial	Experienced	Viremic	DTG + 2 NRTIs	6
Wijting (2017) [12]	Netherlands	Trial	Experienced	VS	DTG	4
Abdullah (2023) [36]	Nigeria	Cohort	Experienced	Viremic	DTG + 2 NRTIs	1
Khamadi (2023) [42]	Tanzania	Cohort	Experienced	Viremic	DTG + 2 NRTIs	3
Oldenbuettel (2017) [11]	Germany	Cohort	Experienced	VS	DTG	1
Bowman (2023) [38]	U.K.	Cohort	Experienced	VS	DTG + 3TC	1
Palmier (2023) [44]	Spain	Cohort	Experienced	VS	DTG + 3TC	1
Armenia (2023) [37]	Italy, France	Case Series	Naïve, Experienced	Viremic, VS	Multiple	9
Diaz (2023) [40]	Brazil	Case Series	Naïve	Viremic	DTG + 2 NRTIs	4
Frange (2021) [23]	France	Case Series	Experienced	Viremic	DTG + 2 NRTIs	1
Gil (2022) [27]	Spain	Case Series	Naïve, Experienced	Not reported	DTG + 2 NRTIs	5
Kamori (2023) [41]	Tanzania	Case Series	Experienced	Viremic	DTG + 2 NRTIs	3
Lepik (2017) [10]	Canada	Case Series	Naïve, Experienced	Viremic	DTG + 2 NRTIs	3
Schramm (2022) [31]	Malawi	Case Series	Experienced	Viremic	DTG + 2 NRTIs	2
Seatla (2021) [24]	Botswana	Case Series	Experienced	Viremic	DTG + 2 NRTIs	3
Van Oosterhout (2022) [34]	Malawi	Case Series	Naïve, Experienced	Viremic, Not reported	DTG + 2 NRTIs	8
Ahmed (2019) [19]	East Africa	Case Report(s)	Experienced	Viremic	DTG + DRV	1
Botha (2022) [26]	South Africa	Case Report(s)	Experienced	Viremic	DTG + 2 NRTIs	1
Cardoso (2018) [14]	Portugal	Case Report(s)	Experienced	Viremic	DTG + 2 NRTIs	2
Cochrane (2018) [15]	U.K.	Case Report(s)	Experienced	Viremic	DTG + 2 NRTIs	1
Fulcher (2018) [16]	U.S.	Case Report(s)	Naïve	Viremic	DTG + 2 NRTIs	1
Lubke (2019) [21]	Germany	Case Report(s)	Naïve	Viremic	DTG + 2 NRTIs	1
M. Chirimuta (2022) [28]	Zimbabwe	Case Report(s)	Experienced	VS, Not reported	DTG + 2 NRTIs	2
Mahomed (2020) [22]	South Africa	Case Report(s)	Experienced	Viremic	DTG + 2 NRTIs	1
Malinga (2023) [43]	South Africa	Case Report(s)	Experienced	Viremic	DTG + 2 NRTIs	1
Pena Lopez (2018) [17]	Spain	Case Report(s)	Naïve	Viremic	DTG + 2 NRTIs	1
Revollo (2022) [30]	Spain	Case Report(s)	Experienced	VS	DTG + 3TC	1
van Kampen (2022) [33]	Netherlands	Case Report(s)	Experienced	Viremic	DTG + 2 NRTIs	1

Footnote: ^1^ For the SAILNG trial published by Cahn, three of the five reports of emergent resistance were presented at a scientific meeting [63]. ^2^ The studies published by Turkova, Vavro, and Khamadi included children. ^3^ The study by Armenia included four persons who received DTG plus 2 NRTIs, two who received DTG monotherapy, and one each who received DTG plus 3TC, DTG plus RPV, and DTG plus ritonavir-boosted darunavir. The mutations associated with each sample from each study are available in the Supplementary Table S1. Abbreviations: VL (virus load), VS (virologically suppressed), VF (virological failure), NRTIs (nucleoside RT inhibitors), OBR (optimized background regimen).

**Table 2:** Numbers of PLWH with Emergent Major INSTI-Associated DRMs According to ART history, Virus Load (VL) Status, and ART Regimen.

ART History	VL Status	ART Regimen	# with DRMs
Naïve	Viremic	DTG + 2 NRTIs	13
Naïve	Viremic	DTG + 3TC	2
Experienced	Viremic	DTG + 2 NRTIs	49
Experienced	Viremic	DTG + OBR	5
Experienced	Viremic	DTG + b/DRV	1
Experienced	VS	DTG	11
Experienced	VS	DTG + 2 NRTIs	4
Experienced	VS	DTG + 3TC	3
Experienced	VS	DTG + RPV	1
Experienced	Uncertain	DTG + 2 NRTIs	9
Experienced	Uncertain	DTG + 3TC	1

Footnote: Abbreviations: VL (Virus load), VS (virologically suppressed), NRTIs (nucleoside RT inhibitors), OBR (optimized background regimen), b/DRV (ritonavir-boosted darunavir).

**Table 3:** Positive and Negative Correlations Involving Signature DTG-Selected DRMs ^1^.

DRM A	DRM B	A and B	A Alone	B Alone	Neither	Spearman Rho	*p* Value
*Positively correlated DRMs ^2^*							
G140S	Q148H	3	1	0	95	0.86	<0.001
G140A	Q148K	2	2	2	93	0.48	<0.001
G140A	Q148R	2	2	3	92	0.42	<0.001
T66A	G118R	5	0	23	70	0.37	<0.001
S147G	N155H	4	4	9	82	0.32	0.001
E157Q	R263K	7	0	40	52	0.29	0.004
S147G	Q148R	2	6	3	88	0.27	0.007
G118R	E138K	8	17	8	62	0.24	0.02
L74M	G118R	2	0	25	70	0.23	0.02
G140S	N155H	2	2	11	84	0.22	0.03
T97A	N155H	2	2	11	84	0.22	0.03
A49G	R263K	4	0	43	52	0.22	0.03
L74I	G118R	3	1	25	69	0.21	0.04
***Negatively correlated DRMs ^3^***							
G118R	R263K	3	24	42	28	−0.44	<0.001
E138K	R263K	2	16	43	36	−0.34	<0.001
Q148KRH	R263K	0	11	46	41	−0.33	<0.001
N155H	R263K	2	11	45	41	−0.25	0.01
G118R	N155H	0	29	13	57	−0.25	0.01
T66I	R263K	0	6	47	46	−0.24	0.02
G118R	Q148KRH	0	28	11	59	−0.23	0.03

Footnote: ^1^ DRMs present as part of mixtures were excluded from analysis. ^2^ Only those pairs present in ≥2 PLWH were analyzed. ^3^ For this analysis Q148H/K/R were pooled.

**Table 4:** Drug Susceptibility Results of HIV-1 Isolates Containing a Single Signature DTG-associated DRM According to the Number of Additional INSTI-associated DRMs.

Signature DRM	# Additional DRMs	# Results	Median Fold Reduced Susceptibility	IQR	Range
G118R	0	2	18.8	14–23	9.6–28
	1	7	22	11–29	7.2–30
	≥2	5	16	13–22	8.0–52
R263K	0	7	2.0	1.8–2.2	1.5–3.3
	1	5	2.1	1.7–4.2	1.3–7.0
	≥2	1	6.3	6.3	6.3
N155H	0	8	1.4	1.2–1.6	1.1–2.1
	1	14	1.7	1.5–2.0	1.1–3.5
	≥2	8	3.1	1.9–24	1.5–68
Q148H/R/K	0	11	0.8	0.7–1.1	0.4–1.6
	1	44	3.4	1.9–5.5	0.5–17
	≥2	27	8.8	3.5–15	0.6–186

## Data Availability

All of the data are available in the two supplementary files.

## Supplementary Materials

The following supporting information can be downloaded at: https://www.mdpi.com/article/10.3390/v15091932/s1, Supplementary Table S1; Supplementary Table S2.
